# The unfolded protein response of the endoplasmic reticulum protects *Caenorhabditis elegans* against DNA damage caused by stalled replication forks

**DOI:** 10.1093/g3journal/jkae017

**Published:** 2024-01-24

**Authors:** Jiaming Xu, Brendil Sabatino, Junran Yan, Glafira Ermakova, Kelsie R S Doering, Stefan Taubert

**Affiliations:** Graduate Program in Cell & Developmental Biology, The University of British Columbia, 950 W 28th Ave, Vancouver, BC V5Z 4H4, Canada; Centre for Molecular Medicine and Therapeutics, The University of British Columbia, 950 W 28th Ave, Vancouver, BC V5Z 4H4, Canada; British Columbia Children’s Hospital Research Institute, 950 W 28th Ave, Vancouver, BC V5Z 4H4, Canada; Centre for Molecular Medicine and Therapeutics, The University of British Columbia, 950 W 28th Ave, Vancouver, BC V5Z 4H4, Canada; British Columbia Children’s Hospital Research Institute, 950 W 28th Ave, Vancouver, BC V5Z 4H4, Canada; Department of Medical Genetics, The University of British Columbia, 950 W 28th Ave, Vancouver, BC V5Z 4H4, Canada; Centre for Molecular Medicine and Therapeutics, The University of British Columbia, 950 W 28th Ave, Vancouver, BC V5Z 4H4, Canada; British Columbia Children’s Hospital Research Institute, 950 W 28th Ave, Vancouver, BC V5Z 4H4, Canada; Edwin S.H. Leong Centre for Healthy Aging, The University of British Columbia, 117-2194 Health Sciences Mall, Vancouver, BC V6T 1Z3, Canada; Department of Medical Genetics, The University of British Columbia, 950 W 28th Ave, Vancouver, BC V5Z 4H4, Canada; Centre for Molecular Medicine and Therapeutics, The University of British Columbia, 950 W 28th Ave, Vancouver, BC V5Z 4H4, Canada; British Columbia Children’s Hospital Research Institute, 950 W 28th Ave, Vancouver, BC V5Z 4H4, Canada; Edwin S.H. Leong Centre for Healthy Aging, The University of British Columbia, 117-2194 Health Sciences Mall, Vancouver, BC V6T 1Z3, Canada; Department of Medical Genetics, The University of British Columbia, 950 W 28th Ave, Vancouver, BC V5Z 4H4, Canada; Centre for Molecular Medicine and Therapeutics, The University of British Columbia, 950 W 28th Ave, Vancouver, BC V5Z 4H4, Canada; British Columbia Children’s Hospital Research Institute, 950 W 28th Ave, Vancouver, BC V5Z 4H4, Canada; Edwin S.H. Leong Centre for Healthy Aging, The University of British Columbia, 117-2194 Health Sciences Mall, Vancouver, BC V6T 1Z3, Canada; Department of Medical Genetics, The University of British Columbia, 950 W 28th Ave, Vancouver, BC V5Z 4H4, Canada; Graduate Program in Cell & Developmental Biology, The University of British Columbia, 950 W 28th Ave, Vancouver, BC V5Z 4H4, Canada; Centre for Molecular Medicine and Therapeutics, The University of British Columbia, 950 W 28th Ave, Vancouver, BC V5Z 4H4, Canada; British Columbia Children’s Hospital Research Institute, 950 W 28th Ave, Vancouver, BC V5Z 4H4, Canada; Edwin S.H. Leong Centre for Healthy Aging, The University of British Columbia, 117-2194 Health Sciences Mall, Vancouver, BC V6T 1Z3, Canada; Department of Medical Genetics, The University of British Columbia, 950 W 28th Ave, Vancouver, BC V5Z 4H4, Canada

**Keywords:** UPR, BiP, DNA replication, DNA damage, primase

## Abstract

All animals must maintain genome and proteome integrity, especially when experiencing endogenous or exogenous stress. To cope, organisms have evolved sophisticated and conserved response systems: unfolded protein responses (UPRs) ensure proteostasis, while DNA damage responses (DDRs) maintain genome integrity. Emerging evidence suggests that UPRs and DDRs crosstalk, but this remains poorly understood. Here, we demonstrate that depletion of the DNA primases *pri-1* or *pri-2*, which synthesize RNA primers at replication forks and whose inactivation causes DNA damage, activates the UPR of the endoplasmic reticulum (UPR-ER) in *Caenorhabditis elegans*, with especially strong activation in the germline. We observed activation of both the inositol-requiring-enzyme 1 (*ire-1*) and the protein kinase RNA-like endoplasmic reticulum kinase (*pek-1*) branches of the (UPR-ER). Interestingly, activation of the (UPR-ER) output gene heat shock protein 4 (*hsp-4*) was partially independent of its canonical activators, *ire-1* and X-box binding protein (*xbp-1*), and instead required the third branch of the (UPR-ER), activating transcription factor 6 (*atf-6*), suggesting functional redundancy. We further found that primase depletion specifically induces the (UPR-ER), but not the distinct cytosolic or mitochondrial UPRs, suggesting that primase inactivation causes compartment-specific rather than global stress. Functionally, loss of *ire-1* or *pek-1* sensitizes animals to replication stress caused by hydroxyurea. Finally, transcriptome analysis of *pri-1* embryos revealed several deregulated processes that could cause (UPR-ER) activation, including protein glycosylation, calcium signaling, and fatty acid desaturation. Together, our data show that the (UPR-ER), but not other UPRs, responds to replication fork stress and that the (UPR-ER) is required to alleviate this stress.

## Introduction

The endoplasmic reticulum (ER) of eukaryotes is a dynamic membrane network required for many cellular processes, and ER homeostasis is critical to cellular and organismal health. ER homeostasis is disturbed by both impaired proteostasis and by ER membrane lipid disequilibrium (Gardner *et al*. 2013; Senft and Ronai 2015; Xu and Taubert 2021; Celik *et al*. 2023). To ensure ER function and cell viability, a conserved adaptive mechanism has evolved that restores ER homeostasis during stress: the ER unfolded protein response (UPR-ER) (Walter and Ron 2011; Gardner *et al*. 2013; Senft and Ronai 2015; Hetz *et al*. 2020; Xu and Taubert 2021). In higher eukaryotes, the UPR-ER consists of 3 parallel ER stress sensing and transducing branches: the inositol-requiring-enzyme 1 (IRE1/IRE-1, also known as ER to nucleus signaling 1 or ERN1 in mammals) branch (Adams *et al*. 2019), the protein kinase RNA-like ER kinase (PERK/PEK-1, also known as eukaryotic translation initiation factor 2α kinase 3 or EIF2AK3) branch (McQuiston and Diehl 2017), and the activating transcription factor 6 (ATF6/ATF-6) branch (Hillary and FitzGerald 2018). Together, they alleviate ER stress by reprograming transcription and translation to promote protein folding, degradation, and transport, as well as lipid synthesis and remodeling. If ER stress cannot be resolved, the UPR-ER switches from promoting survival and adaptation to triggering apoptosis, ensuring tissue and organism integrity (Walter and Ron 2011; Hetz *et al*. 2020).

Like ER homeostasis, genome integrity is paramount for cellular and organismal health. Cells have to safeguard against DNA damage caused by endogenous and exogenous agents that induce different types of DNA lesions. To repair and mitigate DNA damage, cells activate DNA repair pathways and modulate cell cycle progression and apoptosis, a response collectively known as the DNA damage response (DDR) (Jackson and Bartek 2009; Gartner and Engebrecht 2021; McClure *et al*. 2022). Repairing this damage is critical to ensuring faithful DNA replication and thus cell division and organism development, growth, maintenance, and aging.

Interestingly, there is crosstalk between the UPR-ER and the DDR (González-Quiroz *et al*. 2020; Bolland *et al*. 2021). In yeast, *IRE1* promotes DNA repair via several different pathways, and deletion of *IRE1* sensitizes yeast to genotoxic stress and causes chromosome loss even in unstressed conditions (Henry *et al*. 2010). Moreover, activation of the checkpoint pathway by DNA damage upregulates the key UPR-ER output transcription factor Hac1p (Tao *et al*. 2011). Similarly, in cultured human cell lines, IRE-1 promotes genome integrity through the downstream effector X-box binding protein 1 (XBP1; the human ortholog of Hac1p), which directly regulates several DDR pathways, and through noncanonical regulated IRE-1-dependent decay (RIDD) of mRNA (Dufey *et al*. 2020). Consequently, loss of XBP1 correlates with increased DNA damage (Acosta-Alvear *et al*. 2007; Argemí *et al*. 2017; Lyu *et al*. 2019; González-Quiroz *et al*. 2020). Moreover, DNA damaging agents such as camptothecin and ionizing radiation trigger UPR-ER activation in cancer cell lines through the conserved DDR sensor (Ataxia-telangiectasia mutated; Hotokezaka *et al*. 2020), and the genome integrity regulator p53 mediates ER structure remodeling in chemically induced genotoxicity (Zheng *et al*. 2018). The UPR-ER, especially the IRE-1 branch, is therefore both activated by DNA damage and functionally required to repair such damage.

Whether integration of the DDR and the UPR-ER also occurs in animals, and how different tissues respond in this context, is less well understood. In the nematode worm *Caenorhabditis elegans*, DNA damage in the adult germline promotes stress resistance in the postmitotic soma via MAP kinase signaling, innate immune responses, and the ubiquitin proteasome system (Ermolaeva *et al*. 2013), which, like the UPR-ER, maintains proteostasis (Papaevgeniou and Chondrogianni 2014; Zhang *et al*. 2022). Moreover, *C. elegans xbp-1* is required to express DNA repair genes (Shen *et al*. 2005). Recently, increased stress resistance to the ER stressors dithiothreitol (DTT) and tunicamycin was observed in *C. elegans* exposed to UV, which increased the activity of the IRE-1–XBP-1 branch by elevating the levels of unsaturated phosphatidylcholine (Deng *et al*. 2021), a key ER membrane lipid (van Meer *et al*. 2008). However, this study predominantly analyzed DDR and UPR-ER signaling in the *glp-1* mutant, which lacks a germline, is long-lived and stress resistant, and shows dysregulation of DDR genes (Arantes-Oliveira *et al*. 2002; Boyd *et al*. 2010; TeKippe and Aballay 2010; Ratnappan *et al*. 2014; Goh *et al*. 2018). The interaction of the UPR-ER and the DDR in wild-type animals with an intact germline, which is the primary tissue of active DNA repair, therefore remains incompletely understood.

In a screen for genes whose inactivation causes UPR-ER activation in wild-type *C. elegans*, we identified two DNA primase genes (Ho *et al*. 2020). Eukaryotic primase complexes synthesize short RNA primers required for initiating lagging strand DNA replication and also contribute to DNA repair and possibly transcription (Guilliam *et al*. 2015; Yoon *et al*. 2018). Abnormal primase function causes stalled replication forks, leading to DNA damage and genome instability. This, therefore, provided us with the opportunity to study the relationship between primase function, replication stress, and the UPR-ER in animals with an intact germline. Here, we show that primase inactivation and UV–C irradiation activate both the IRE-1 and the PEK-1 branches of the UPR-ER, with stronger induction in the germline than in the soma. Interestingly, activation of the heat shock protein 4 (*hsp-4*) gene, which canonically requires the IRE-1–XBP-1 axis, required *atf-6* in the germline, suggesting differential regulatory mechanisms. We further found that primase inactivation selectively activated the UPR-ER, but not the cytosolic or mitochondrial UPRs, arguing for a specific role of the UPR-ER in maintaining genome integrity. We also showed that loss of *ire-1* or *pek-1* sensitizes *C. elegans* to replication stress, showing that the UPR-ER is functionally protective. RNA-sequencing (RNA-seq) analysis revealed several pathways comprising both proteostasis and lipidostasis that could underlie UPR-ER activation following replication stress. Collectively, our data show that the UPR-ER plays important roles in ensuring genome integrity in *C. elegans*.

## Materials and methods

### Worm strains

The following worm strains were used: N2 wild-type, SJ4005  *zcIs4 [hsp-4p::gfp] V* (Calfon *et al*. 2002), SJ17  *xbp-1(zc12) III; zcIs4 [hsp-4p::gfp] V* (Calfon *et al*. 2002), SJ30  *ire-1(zc14) II; zcIs4 [hsp-4p::gfp] V* (Calfon *et al*. 2002), SJ4100  *zcIs13 [hsp-6p::gfp] V* (Yoneda *et al*. 2004), TJ375  *gpIs1 [hsp-16.2p::gfp]* (Henderson and Johnson 2001), *xbp-1(tm2482) III* (Richardson *et al*. 2011), RB545  *pek-1(ok275) X* (Consortium 2012), RB925  *ire-1(ok799) II* (Consortium 2012), RB772  *atf-6(ok551) X* (Consortium 2012), PHX2824 *hsp-4::gfp(syb2824) II* (generated by SunyBiotech Co., Ltd., Fujian, China), and STE142 *hsp-4::gfp(syb2824) II*;*atf-6(ok551) X* (this study). All strains were backcrossed 6 times to the laboratory N2 wild-type background before use.

### Worm growth conditions

We cultured *C. elegans* strains at 20°C on nematode growth medium (NGM)-lite agar plates with *E. coli*  OP50 as food source, except for RNA interference (RNAi), for which HT115 strain was used (Ho *et al*. 2020). To developmentally synchronize worm populations, gravid adult worms were treated with alkaline sodium hypochlorite solution to extract embryos, which were washed twice with M9, and then plated onto an unseeded NGM-lite plate to allow hatching overnight. When imaging worms, adult worms were bleached >10 minutes until autofluorescent mother bodies disappeared. The resulting synchronized L1 larvae were transferred onto OP50 NGM-lite plates or RNAi plates (NGM-lite plates containing 25 μg/mL carbenicillin (BioBasic CDJ469), 2 mM Isopropyl ß-D-1-thiogalactopyranoside (Santa Cruz sc-202185B), and 12.5 μg/mL tetracycline (BioBasic TB0504)). RNAi plates were seeded twice with the appropriate HT115 RNAi bacteria (Ahringer library, Source BioScience); RNAi clones were sequenced prior to use to ensure construct identity. Synchronized L1 worms were placed on RNAi plates and grown until they reached the desired developmental stage.

### Differential interference contrast and fluorescence microscopy

Worms were mounted onto 2% (w/v) agarose pads containing a drop of 20 mM sodium azide (NaN_3_) for microscopy. Eggs were picked from plates onto 2% (w/v) agarose pads containing a drop of M9 for microscopy. Worms were imaged using differential interference contrast (DIC) and fluorescence optics through a CoolSnap HQ camera (Photometrics, Tucson, AZ, USA) on a Zeiss Axioplan 2 compound microscope (Carl Zeiss Microscopy, Thornwood, NY, USA). All GFP images were taken at the same exposure time (300 ms). Using the ImageJ software, the images in the GFP channel were adjusted to the same brightness (maximum display value = 4095, minimum = 201; these parameters were applied to all GFP images used for quantification in this study) and contrast levels for subsequent display and quantification purposes. Analysis of overall fluorescence intensity of individual worms was performed by tracing the outline of the worms on the corresponding DIC images, and then normalizing for area and background fluorescence, as described (Shomer *et al*. 2019).

### Protein extraction and immunoblots

Whole-worm protein extracts were generated by sonication in radioimmunoprecipitation assay (RIPA) lysis buffer with cOmpleteTM Protease Inhibitor Cocktail (Roche #4693116001) and β-glycerophosphate (Sigma-Aldrich G6251). Protein concentrations were determined using the reducing agent and detergent compatible (RCDC) protein assay kit (Bio-Rad #500-0121), and sodium dodecyl-sulfate polyacrylamide gel electrophoresis (SDS-PAGE) analysis and immunoblotting were performed as described (Hou *et al*. 2014), using anti-Ser51-Phospho-eIF2α rabbit antibody (Cell Signaling Technologies #9721), anti-α-tubulin mouse antibody (Sigma #T9026), and anti-rabbit HRP-conjugated (New England Biolabs [NEB] #7074) and anti-mouse HRP-conjugated (Cell Signaling Technologies #7076) secondary antibodies. Detection was done using ECL (Pierce #32109).

### Exposure to genotoxic agents

For UV–C exposure, synchronized populations of day-1 adult *C. elegans* were placed on NGM-lite plates seeded with a thin layer of OP50. Uncovered NGM-lite plates were then placed in a Stratalinker 2400 UV Crosslinker (Stratagene) and irradiated with wavelength 254 nm light at 400 J/m^2^. After 24 h of recovery at 20°C, worms and embryos were mounted and imaged.

For hydroxyurea (HU) exposure L1 recovery experiments, age-synchronized L1 populations were grown for 72 h on NGM-lite or on NGM-lite containing either 5 or 10 mM HU (Sigma-Aldrich H8627). Then, worms were transferred to OP50-seeded NGM-lite plates for recovery and egg laying. After 4 h, the number of eggs was counted for each genotype and condition, as indicated.

For HU exposure L4 recovery experiments, synchronized L1 worms were grown for 48 h. Then, age-synchronized L4 populations were transferred to and maintained on either NGM-lite plates or NGM-lite plates containing 20 mM HU for 24 h. Then, adult worms were transferred to OP50 plates for recovery and egg laying. After 4 h, the number of eggs for each genotype and condition was counted.

To measure developmental rate, synchronized L1 populations were grown for 48 h on NGM-lite plates containing DMSO vehicle or 15 mM HU. Then, the number of L4 or older worms and the total number of worms were counted for each genotype and condition.

For body size quantification, synchronized L1 stage worms were grown for 72 h on NGM-lite plates containing DMSO or 15 mM HU, before >10 worms for each genotype and condition were imaged.

### RNA-Sequencing and data analysis

Synchronized L1 N2 worm populations fed empty vector (EV) or *pri-1* RNAi were grown for 96 h at 20°C and allowed to lay eggs. Plates were washed with M9 twice to remove adults and hatched worms before eggs were harvested with a cell scraper. The collected eggs were washed twice with M9 to remove bacteria, and then flash-frozen in an ethanol-dry ice bath. For total RNA extraction, eggs were thawed in Trizol and sonicated. Total RNA was extracted using Trizol and 1-bromo-3-chloropropane, as described (Doering *et al*. 2022). RNA integrity and quantity were assessed on an Agilent Technologies 2100 Bioanalyzer System.

Library preparation and sequencing was performed by The Center for Applied Genomics, SickKids, Toronto, ON (http://www.tcag.ca). Briefly, RNA was prepared for sequencing using the NEBNext Ultra II Directional RNA Library Prep Kit for Illumina (NEB #E7760). Sequencing was performed on an Ilumina NovaSeq 6000 instrument equipped with an S4 flow cell generating 150 bp paired-end reads. Low quality reads and adapter sequences were trimmed using Trimmomatic 0.36 (Bolger *et al*. 2014) with parameters LEADING:3 TRAILING:3 SLIDINGWINDOW:4:15 MINLEN:36. The trimmed reads were quantified to the *C. elegans* Ensembl transcriptome build WBcel235 using Salmon ver1.4.0 (Patro *et al*. 2017) with the parameters -l A -p 8 –gcBias –validateMappings. Then, transcript-level read counts were imported into R and summed into gene-level read counts using tximport (Soneson *et al*. 2016) (genes listed in Supplementary Table 1). Genes not expressed at a level greater than 10 reads in at least 3 of the samples were excluded from further analysis. Differential expression analysis was performed with quasi-likelihood F-test with the generalized linear model (GLM) approach in edgeR (Robinson *et al*. 2010). Genes with *P*-value <0.05 and False Discovery Rate < 0.05 were considered differentially expressed. RNA-seq data were deposited in the Gene Expression Omnibus under the accession number GSE225569. Gene set enrichment analysis (GSEA) using either the biological process (BP (Chagoyen and Pazos 2010)) or the Kyoto encyclopedia of genes and genomes (KEGG (Kanehisa 1997; Kanehisa *et al*. 2020)) as underlying databases was performed with eVITTA (Cheng *et al*. 2021), using *P*val < 0.05 and *P*adj < 0.25 as cutoffs.

### Statistical analysis

*P* values were calculated using 2-tailed Student's t-tests, Welch's t-tests, 1-way ANOVA tests, or 2-way ANOVA tests using GraphPad Prism 9 or 10, as reported in the figure legends. Scatter plots were generated in GraphPad Prism 9 or 10. Error bars denote standard deviation; the number of independent experiments performed and number of animals studied are indicated in the figure legends.

## Results

### Knockdown of the *C. elegans* primase genes *pri-1* or *pri-2* activates the *ire-1* branch of the UPR-ER in embryos

**Fig. 1.**
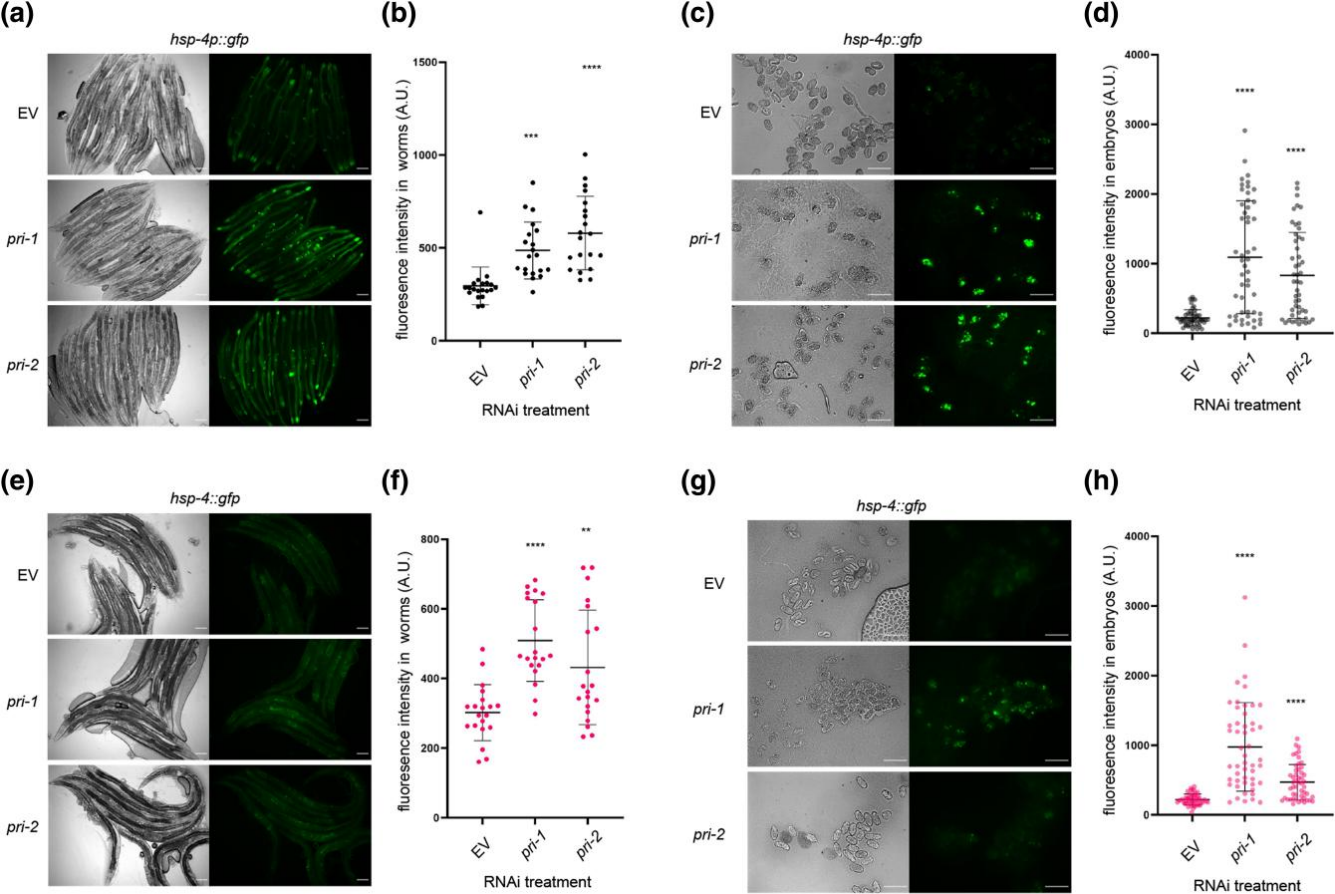
*pri-1* or *pri-2* knockdown induces IRE-1 branch activity in the soma and embryos of *C. elegans*. a and b) The figure shows representative micrographs (a) and whole-worm GFP quantification (b) of *hsp-4p::gfp* adult worms fed EV, *pri-1*, or *pri-2* RNAi (*n* = 3 experiments totaling >20 animals per RNAi treatment). c and d) The figure shows representative micrographs (c) and GFP quantification (d) of F1 embryos laid by *hsp-4p::gfp* adult worms fed EV, *pri-1*, or *pri-2* RNAi (*n* = 3 experiments totaling >50 embryos per RNAi treatment). e and f) The figure shows representative micrographs (e) and whole-worm GFP quantification (f) of *hsp-4::gfp* adult worms fed EV, *pri-1*, or *pri-2* RNAi (*n* = 2 experiments totaling >20 animals per RNAi treatment). g and h) The figure shows representative micrographs (g) and GFP quantification (h) of embryos laid by *hsp-4::gfp* adult worms fed EV, *pri-1*, or *pri-2* RNAi (*n* = 2 experiments totaling >50 embryos per treatment). In all micrographs, the scale bar represents 100 *μ*m. In dot plots, each dot represents the signal detected in one individual worm or embryo; the error bars represent standard deviation. Statistical analysis for b, d, f, h: **P < 0.01, ***P < 0.001, ****P < 0.0001 vs. EV RNAi-treated control (Brown–Forsythe and Welch ANOVA test corrected for multiple comparisons using the Dunnett T3 method).

We previously showed that RNAi against the 2 DNA primase subunit genes of *C. elegans*, *pri-1* or *pri-2*, caused activation of the UPR-ER (Ho *et al*. 2020). To validate this finding, we quantified the induction of *hsp-4p::gfp*, a widely used transcriptional reporter for the ER stress-inducible, *ire-1*- and *xbp-1*-activated *hsp-4* gene promoter (Calfon *et al*. 2002; Hou *et al*. 2014; Ho *et al*. 2020). We found that *pri-1* or *pri-2* RNAi induced *sp-4p::gfp* fluorescence in the worm soma ∼1.5–2-fold (Fig. 1, a and b). Interestingly, we observed a larger increase in *hsp-4p::gfp* activity (∼4-fold) in F1 embryos from RNAi-fed P0 adults (Fig. 1, c and d). This phenotype manifests despite the fact that F1 eggs from *pri-1* or *pri-2* RNAi-treated P0 adults never hatch, but rather arrest at the early embryogenesis/pre-morphogenetic stage due to persisting replication fork stalling, which causes double-stranded DNA breaks (DSBs) (Zeman and Cimprich 2014). UPR-ER activation in the F1 generation by *pri-1* or *pri-2* RNAi in P0 suggested a link between the UPR-ER and genotoxic stress in embryos.

To confirm activation of the endogenous UPR-ER by *pri-1* or *pri-2* RNAi, we studied a genome-edited strain wherein the 3′ end of the *hsp-4* coding sequence is tagged with *gfp*, resulting in a C-terminal HSP-4::GFP fusion protein (hereafter referred to as *hsp-4::gfp*). To validate this strain, we examined GFP intensity following proteotoxic stress by tunicamycin and lipotoxic stress by *mdt-15* RNAi, both established UPR-ER inducers (Calfon *et al*. 2002; Hou *et al*. 2014). As expected, we observed elevated GFP intensity in *hsp-4::gfp* worms challenged with tunicamycin or *mdt-15* RNAi (Supplementary Fig. 1), suggesting that this strain faithfully reports on the regulation of endogenous *hsp-4* by different stresses. Next, we treated the *hsp-4::gfp* reporter strain with *pri-1* or *pri-2* RNAi and studied GFP fluorescence in the soma of P0 adult worms and in F1 embryos. We observed strong induction of endogenous HSP-4::GFP in F1 embryos, and weaker induction in P0 somatic cells (Fig. 1, e–h). These data suggest that loss of primase function and subsequent replication defects trigger UPR-ER activation in embryos and in somatic cells of *C. elegans*, with stronger induction in embryos.

### Knockdown of *pri-1* or *pri-2* induces embryonic *hsp-4* partially independently of *ire-1* and *xbp-1*

**Fig. 2.**
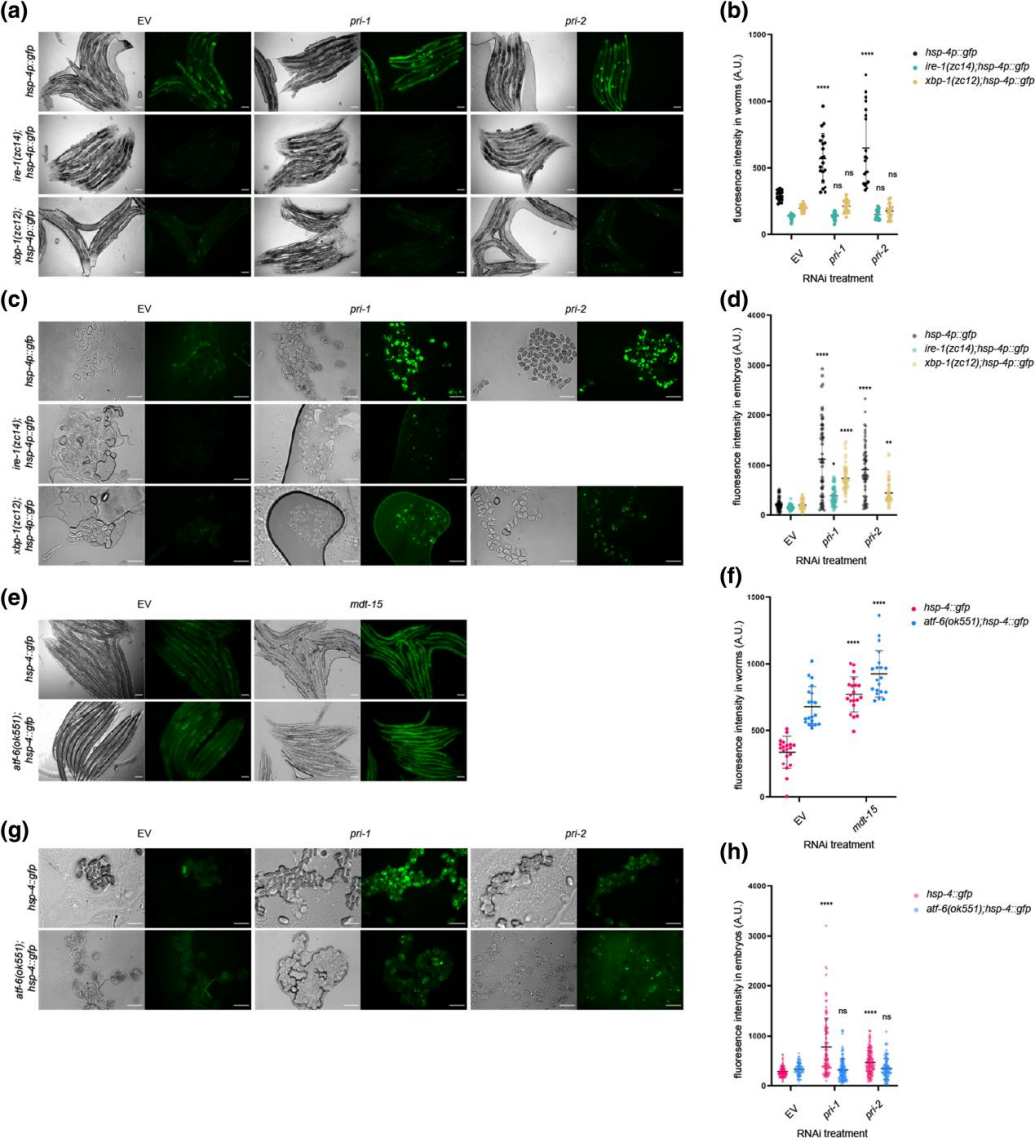
Activation of *hsp-4* by *pri-1* or *pri-2* RNAi requires *ire-1, xbp-1*, and *atf-6*. a and b) The figure shows representative micrographs (a) and whole-worm GFP quantification (b) of *hsp-4p::gfp*, *ire-1(zc14);hsp-4p::gfp*, and *xbp-1(zc12);hsp-4p::gfp* adult worms fed EV, *pri-1*, or *pri-2* RNAi (*n* = 3 experiments totaling >20 individual animals per RNAi treatment). c and d) The figure shows representative micrographs (c) and GFP quantification (d) of embryos laid by *hsp-4p::gfp*, *ire-1(zc14);hsp-4p::gfp*, and *xbp-1(zc12);hsp-4p::gfp* adult worms fed EV, *pri-1*, or *pri-2* RNAi (*n* = 3 experiments totaling >50 individual embryos per RNAi treatment; note that *pri-2* caused lethality in this experiment, preventing experimental assessment). e and f) The figure shows representative micrographs (e) and whole-worm GFP quantification (f) of *hsp-4::gfp* and *atf-6 (ok551);hsp-4::gfp* adult worms fed EV or *mdt-15* RNAi (*n* = 3 experiments totaling >20 individual animals per RNAi treatment). g and h) The figure shows representative micrographs (g) and GFP quantification (h) of embryos laid by *hsp-4::gfp* and *atf-6(ok551);hsp-4::gfp* adult worms fed EV, *pri-1*, or *pri-2* RNAi (*n* = 3 experiments totaling >75 individual embryos per treatment). In all micrographs, the scale bar represents 100 *μ*m. In dot plots, each dot represents the signal detected in one individual worm or embryo; the error bars represent standard deviation. Statistical analysis: b, d: ns *P* > 0.05, *P < 0.05, **P < 0.01, ****P < 0.0001, vs. EV RNAi-treated control of the same genotype (2-way ANOVA test corrected for multiple comparisons using Sidak's method).

Canonical *hsp-4* induction requires the transmembrane ER stress sensor *ire-1* and the downstream transcription factor *xbp-1* (Shen *et al*. 2001; Richardson *et al*. 2010). Thus, we studied *hsp-4* induction in *ire-1; hsp-4p::gfp,* and *xbp-1; hsp-4p::gfp* worms treated with *pri-1* or *pri-2* RNAi. Consistent with canonical UPR-ER induction in somatic cells, increased fluorescence in *pri-1-* or *pri-2-*treated worms depended completely on *ire-1* and *xbp-1* (Fig. 2, a and b). By contrast, significant induction of *hsp-4p::gfp* remained in embryos when *ire-1* or *xbp-1* was deleted (Fig. 2, c and d). This suggests that additional genes are required to induce *hsp-4* in embryos experiencing replication stress.

### *atf-6* is required for *hsp-4* induction in *pri-1* or *pri-2* RNAi-treated embryos

In mammals, ATF6 is required for the transcription of *XBP1* mRNA (Yoshida *et al*. 2001; Lee *et al*. 2002). Thus, we tested if *C. elegans atf-6* is required for replication-stress-induced *hsp-4* induction. We crossed the *hsp-4::gfp* translational reporter into a strain bearing the *atf-6(ok551)* null allele, and treated *atf-6; hsp-4::gfp* worms with *pri-1* or *pri-2* RNAi. As a control, we first studied *mdt-15* RNAi, which caused *hsp-4::gfp* induction in the soma despite the *atf-6* mutation (Fig. 2, e and f), suggesting that *hsp-4* induction in somatic tissues does not require *atf-6*; unexpectedly, *atf-6* loss alone induced *hsp-4* expression in the soma (Fig. 2, e and f). Notably, *atf-6* deletion reduced the increased fluorescence in embryos treated with *pri-1* or *pri-2* RNAi (Fig. 2, g and h). This indicates that *hsp-4* induction in *pri-1* or *pri-2* RNAi-treated embryos depends on *atf-6*.

### *pri-1* or *pri-2* knockdown activates the *pek-1* branch of the UPR-ER

**Fig. 3.**
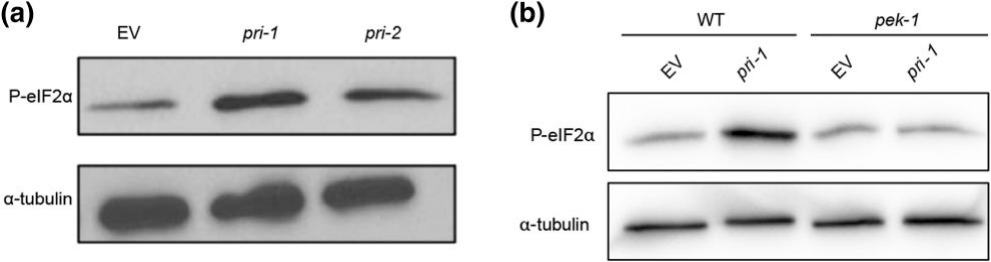
*pri-1* or *pri-2* RNAi activate the PEK-1 branch in the embryos of *C. elegans*. a and b) The immunoblot depicts the levels of phospho-Ser51 eIF2α (P-eIF2α) and α-tubulin in EV, *pri-1*, or *pri-2* RNAi-treated wild-type or *pek-1* mutant worm embryos (*n* = 2–3, for additional repeats, please see Supplementary Fig. 2).

The *C. elegans* UPR-ER also features a branch controlled by the kinase PEK-1, which causes phosphorylation of the eukaryotic translation initiation factor eIF2α and subsequent activation of the transcription factor ATF-4 (McQuiston and Diehl 2017). As a readout of PEK-1 activity, we performed immunoblots on embryos to detect phospho-Ser51 on eIF2α, a marker for activated PEK-1 (Nukazuka *et al*. 2008). We observed increased levels of phospho-Ser51 in *pri-1* or *pri-2* RNAi-treated embryos (Fig. 3a, Supplementary Fig. 2). Critically, this induction was dependent on *pek-1* (Fig. 3b, Supplementary Fig. 2), implicating canonical signaling via this branch of the UPR-ER. Thus, *pri-1* or *pri-2* RNAi activates both the *ire-1* and *pek-1* branches of the UPR-ER in the soma and in the embryos.

### The cytosolic and the mitochondrial UPRs are not substantially induced by *pri-1* or *pri-2* knockdown

**Fig. 4.**
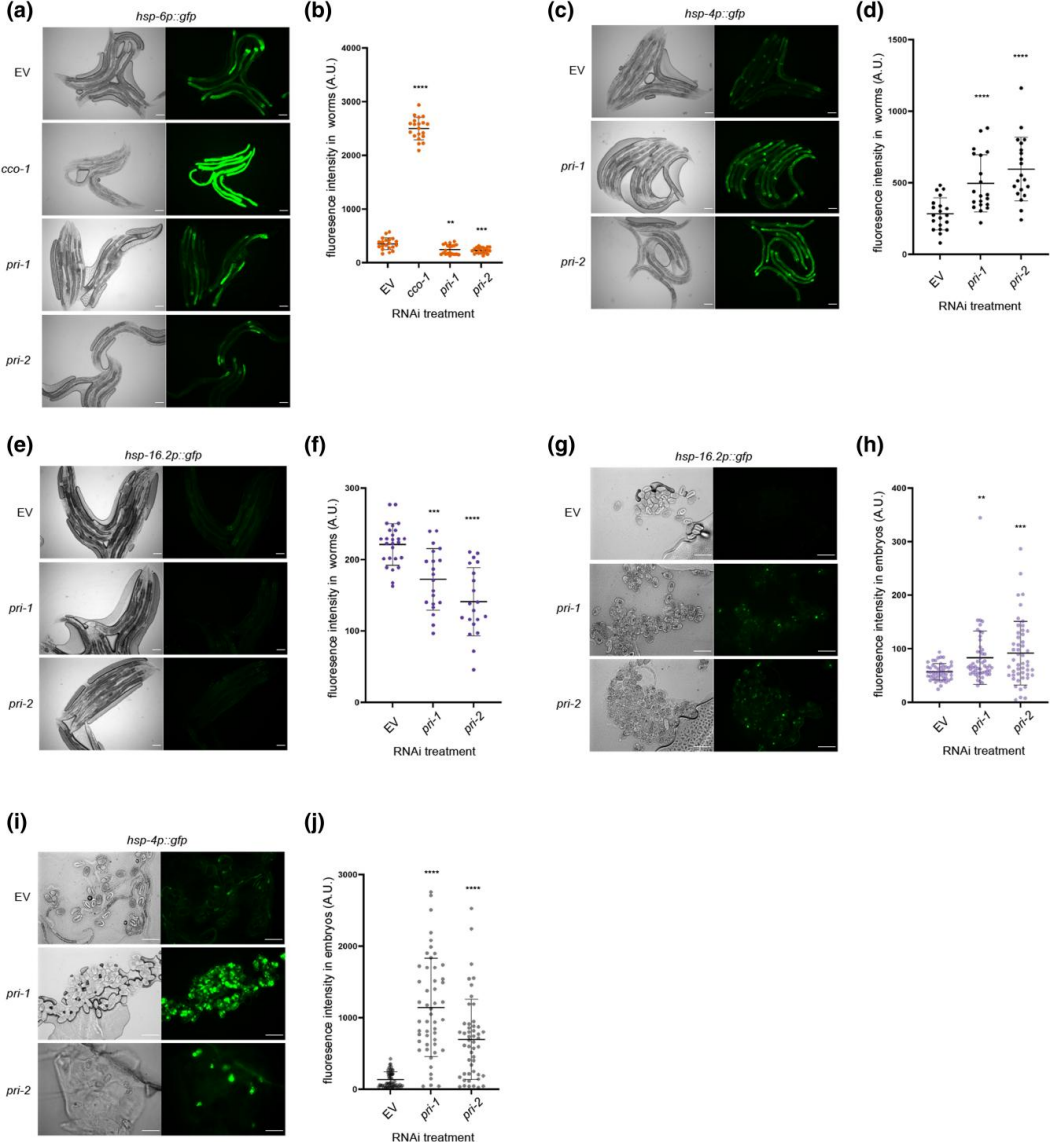
*pri-1* or *pri-2* RNAi preferentially induces the UPR-ER. a and b) The figure shows representative micrographs (a) and whole-worm GFP quantification (b) of *hsp-6p::gfp* adult worms fed EV, *cco-1*, *pri-1*, or *pri-2* RNAi (*n* = 3 experiments totaling >20 individual animals per RNAi treatment). c and d) The figure shows representative micrographs (a) and whole-worm GFP quantification (b) of *hsp-4p::gfp* adult worms fed EV, *pri-1*, or *pri-2* RNAi (*n* = 3 experiments totaling >20 individual animals per RNAi treatment). e and f) The figure shows representative micrographs (e) and whole-worm GFP quantification (f) of *hsp-16.2p::gfp* adult worms fed EV, *pri-1*, or *pri-2* RNAi (*n* = 2 experiments totaling >20 individual animals per RNAi treatment). g and h) The figure shows representative micrographs (g) and GFP quantification (h) of embryos laid by *hsp-16.2p::gfp* adult worms fed EV, *pri-1*, or *pri-2* RNAi (*n* = 3 experiments totaling >50 individual embryos per treatment). i and j) The figure shows representative micrographs (i) and GFP quantification (j) of embryos laid by *hsp-4p::gfp* adult worms fed EV, *pri-1*, or *pri-2* RNAi (*n* = 3 experiments totaling >50 individual embryos per treatment). In all micrographs, the scale bar represents 100 *μ*m. In dot plots, each dot represents the signal detected in one individual worm or embryo; the error bars represent standard deviation. Statistical analysis for b, d, f, h, j: **P < 0.01, ***P < 0.001, ****P < 0.0001 vs. EV RNAi-treated control (Brown–Forsythe and Welch ANOVA test corrected for multiple comparisons using the Dunnett T3 method).

Induction of the UPR-ER due to *pri-1* or *pri-2* RNAi might reflect general protein misfolding in terminally arrested embryos. Thus, we monitored the activity of the cytosolic and mitochondrial UPRs with their well-established reporters, *hsp-16.2p::gfp* and *hsp-6p::gfp*, respectively (Rea *et al*. 2005; Bennett *et al*. 2014). In the worm soma, heat shock strongly induced *hsp-16.2p::gfp* (Supplementary Fig. 3a, b), and positive control *cco-1* RNAi strongly induced *hsp-6p::gfp* (Fig. 4, a and b), as expected (Bennett *et al*. 2014). By contrast, although *pri-1* or *pri-2* RNAi effectively induced *hsp-4p::gfp* (Fig. 4, c and d), it did not induce *hsp-6p::gfp* or *hsp-16.2p::gfp* in somatic tissues; the activity of both reporters was in fact reduced (Fig. 4, a, b, e, and f). Similarly, whereas heat stress strongly (∼5-fold) induced *hsp-16.2p::gfp* throughout the embryo (Supplementary Fig. 3, c and d), *pri-1* or *pri-2* RNAi only weakly (less than 2-fold), albeit still significantly, induced *hsp-16.2p::gfp* fluorescence in the embryos (Fig. 4, g and h), while strongly inducing *hsp-4p::gfp* (Fig. 4, i and j). Thus, *pri-1* or *pri-2* RNAi-induced replication stress appears to predominantly trigger the UPR-ER.

### Inactivation of other polymerase α primase complex genes phenocopies *pri-1* or *pri-2* RNAi

**Fig. 5.**
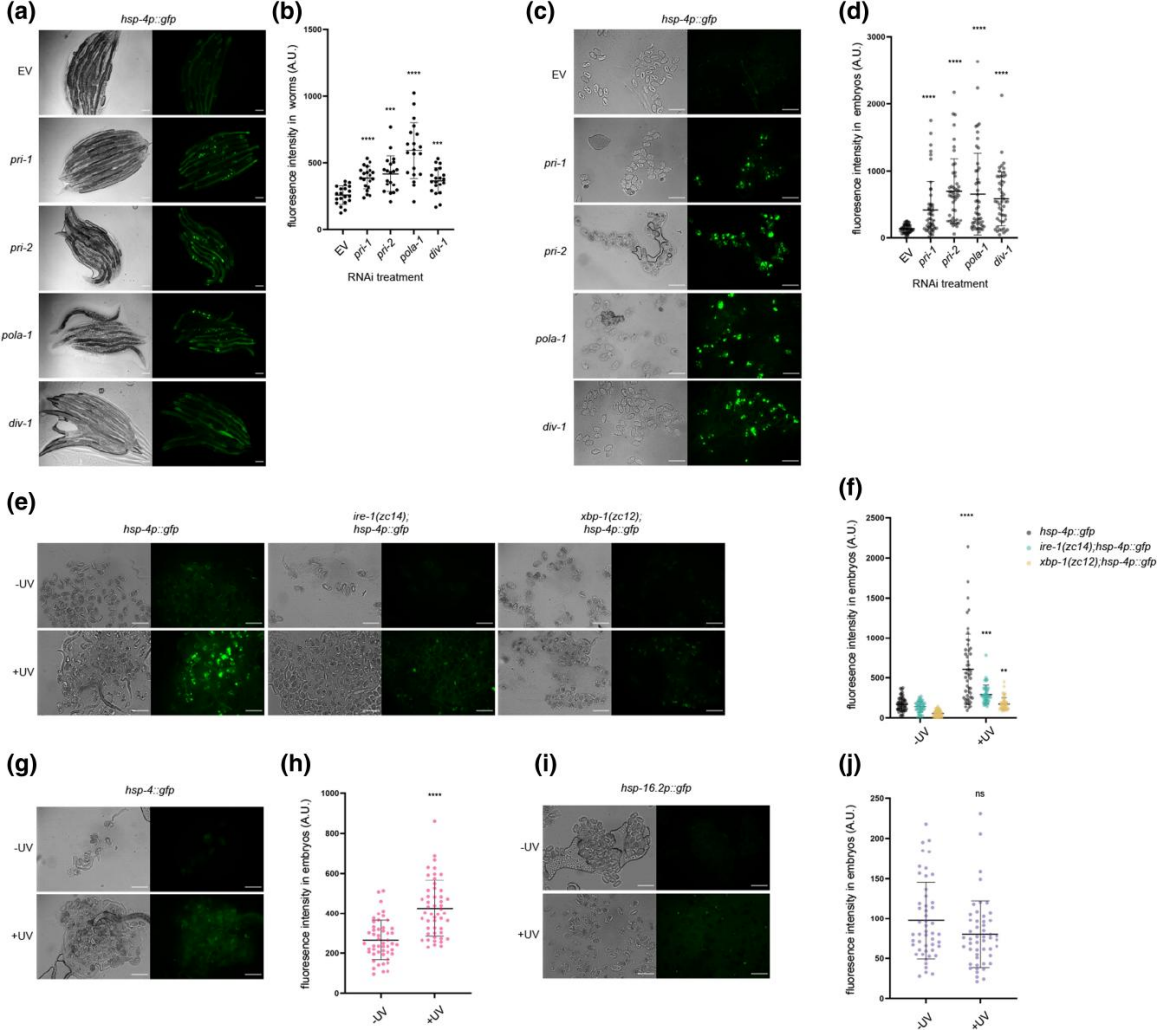
Knockdown of polymerase α primase complex subunits and UV–C irradiation cause UPR-ER activation. a and b) The figure shows representative micrographs (a) and whole-worm GFP quantification (b) of *hsp-4p::gfp* adult worms fed EV, *pri-1*, *pri-2*, *pola-1*, or *div-1* RNAi (*n* = 3 experiments totaling >20 individual animals per RNAi treatment). c, d) The figure shows representative micrographs (c) and GFP quantification (d) of embryos laid by *hsp-4p::gfp* adult worms fed EV, *pri-1*, *pri-2*, *pola-1*, or *div-1* RNAi (*n* = 3 experiments totaling >50 individual embryos per RNAi treatment). e and f) The figure shows representative micrographs (e) and GFP quantification (f) of embryos laid by *hsp-4p::gfp*, *ire-1(zc14);hsp-4p::gfp*, and *xbp-1(zc12);hsp-4p::gfp* adult worms irradiated with 400 J/m^2^ UV–C (*n* = 3 experiments repeats, totaling >50 individual embryos for each sample). g and h) The figure shows representative micrographs (g) and GFP quantification (h) of embryos laid by *hsp-4::gfp* adult worms irradiated with 400 J/m^2^ UV–C (*n* = 3 experiments totaling >50 individual embryos for UV-irradiated and nonirradiated samples). i and j) The figure shows representative micrographs (i) and GFP quantification (j) of embryos laid by *hsp-16.2p::gfp* adult worms irradiated with 400 J/m^2^ UV–C (*n* = 3 experiments totaling >50 individual embryos for UV-irradiated and nonirradiated samples). In all micrographs, the scale bar represents 100 *μ*m. In dot plots, each dot represents the signal detected in one individual worm or embryo; the error bars represent standard deviation. Statistical analysis: b, d: ***P < 0.001, ****P < 0.0001 vs. EV RNAi control (Brown–Forsythe and Welch ANOVA test corrected for multiple comparisons using the Dunnett T3 method); f: **P < 0.01, ***P < 0.001, ****P < 0.0001 vs. nonirradiated embryos of the same genotype (ordinary 2-way ANOVA test corrected for multiple comparisons using Sidak's method); h, j: ns *P* > 0.05, ***P < 0.001, ****P < 0.0001 vs. nonirradiated embryos (Welch's t-test).

Four genes encode *C. elegans* primase complex subunits: the DNA polymerase α catalytic subunit gene *pola-1*, the DNA polymerase α accessory subunit gene *div-1*, and the primase subunit genes *pri-1* and *pri-2* (Guilliam *et al*. 2015; Yoon *et al*. 2018). We asked if RNAi knockdown of *pola-1* or *div-1* phenocopied *pri-1* or *pri-2* RNAi. Indeed, *pola-1* and *div-1* RNAi activated *hsp-4p::gfp* in both the soma of he P0 worms and in F1 embryos, with stronger activation in embryos than in the soma (Fig. 5, a–d). This suggests that UPR-ER induction likely results from replication stress caused by defective polymerase α primase complex function.

### UV–C treatment phenocopies *pri-1* or *pri-2* RNAi

Like replication block, ultraviolet C (UV–C) light induces DSBs if the resulting bipyrimidine photoproducts are not resolved by the nucleotide excision repair (NER) pathway (Stergiou *et al*. 2011). Thus, we tested if UV–C exposure in early embryos phenocopies *pri-1* or *pri-2* RNAi treatment. We irradiated day 1 P0 adult worms with 400J/m^2^ UV–C and studied F1 embryos 24 h thereafter. Like *pri-1* or *pri-2* RNAi, UV–C treatment strongly activated *hsp-4p::gfp* (Fig. 5, e and f), in line with previously published observations (Deng *et al*. 2021). As observed for *pri-1* or *pri-2* RNAi, UV–C-induced *hsp-4p::gfp* upregulation was partially independent of *ire-1* and *xbp-1* in embryos (Fig. 5, e and f). The *hsp-4::gfp* translational reporter was also induced by UV–C (Fig. 5, g and h). By contrast, the cytosolic UPR reporter *hsp-16.2p::gfp* was not activated (Fig. 5, i and j), suggesting that UV–C specifically activates the UPR-ER in embryos. In the soma, UV–C caused ∼2-fold activation of *hsp-16.2p::gfp*, whereas *hsp-4p::gfp*, *hsp-4::gfp*, and *hsp-6p::gfp* were not activated (Supplementary Fig. 4). Collectively, these observations suggest that UV–C irradiation, like *pri-1* or *pri-2* RNAi, primarily activates the UPR-ER, especially in the embryo.

### Inactivating components of the DNA repair machinery does not activate the UPR-ER in somatic cells

The abovementioned data raised the possibility that genotoxic stress in general activates the UPR-ER. To test this hypothesis, we used RNAi to inactivate several DNA repair genes, which should cause increased DNA damage, specifically: *msh-2* (mismatch repair (Degtyareva *et al*. 2002)), *xpf-1* (NER (Saito *et al*. 2009)), *him-1* (a cohesin, whose loss results in chromosomal segregation defects in mitosis and meiosis (Chan *et al*. 2003)), *mus-81* (replicative repair (O’Neil *et al*. 2013)), *dog-1* and *him-6* (whose loss causes formation of R-loops or G4 structures, causing deletions in poly-G tracts and genome instability (Cheung *et al*. 2002; Youds *et al*. 2006)), and *cid-1* (DNA damage checkpoint, whose loss reverts HU-induced developmental arrest and activates *hsp-4p::gfp* (Olsen *et al*. 2006)). Unlike *pri-1* or *pri-2* RNAi, the knockdown of none of these genes activated any UPR-ER reporter in somatic cells (Supplementary Fig. 5). Notably, in our hands, *cid-1* RNAi failed to induce *hsp-4* (Supplementary Fig. 5), possibly because we initiate RNAi in synchronized L1 stage larvae and not in embryos. In embryos, inactivation of *him-1* activated the UPR-ER (Supplementary Fig. 6), but RNAi of the other tested DNA repair genes did not. We conclude that inactivation of DDR and repair machinery genes does not consistently activate the UPR-ER.

### The UPR-ER is not required to protect the germline against HU-induced replication stress

**Fig. 6.**
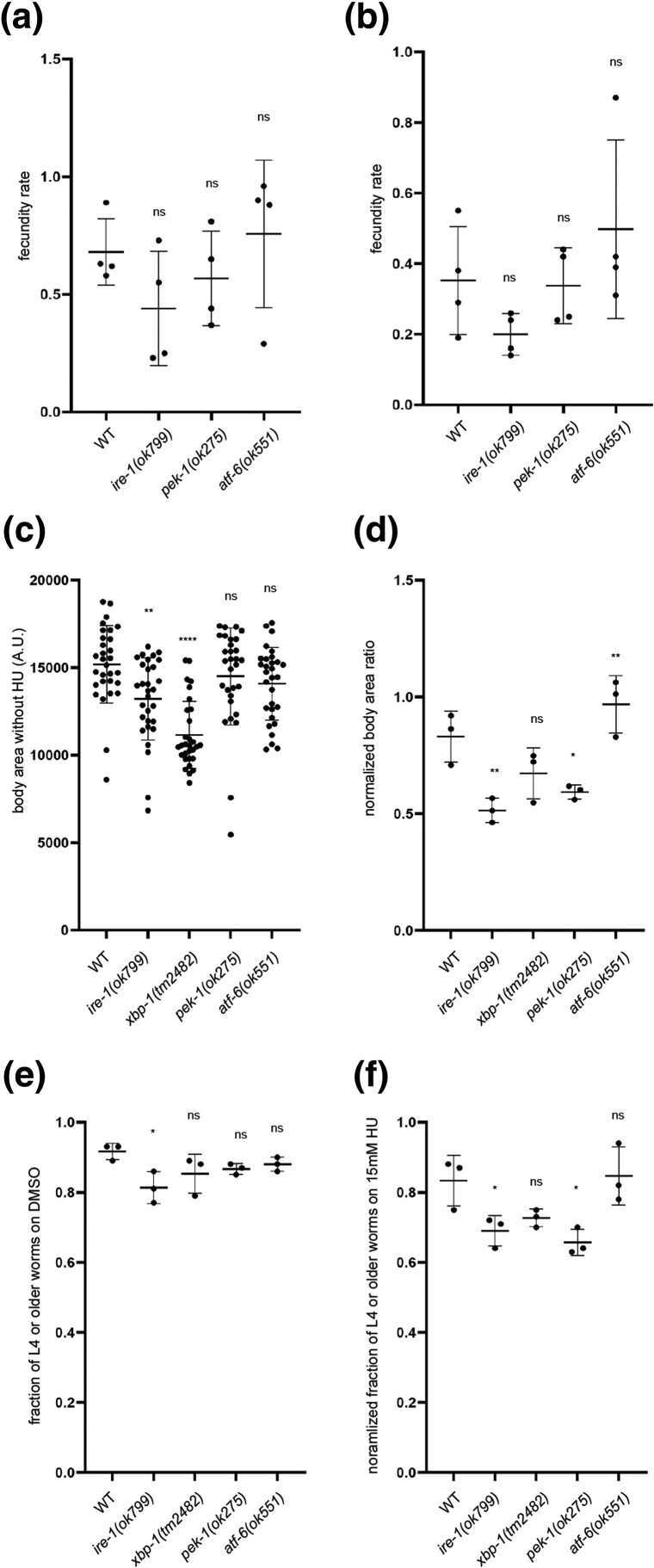
*ire-1* and *pek-1* are required to protect *C. elegans* against HU-induced replication stress. a and b) The graphs show the relative fecundity of wild-type, *ire-1(ok799)*, *pek-1(ok275)*, or *atf-6(ok551)* worms subjected to 20 mM HU for 24 h from late L4 stage (a) or 10 mM HU for 72 h from L1 stage (b). Relative fecundity is calculated as follows: average number of eggs laid by an HU-treated worm during a 4-h postexposure period/average number of eggs laid by an unstressed worm of the same genotype during a 4-h period. c) Body size quantification of wild-type, *ire-1(ok799)*, *xbp-1(tm2482)*, *pek-1(ok275)*, or *atf-6(ok551)* adult worms grown under unstressed conditions (*n* = 3 experiments totaling >30 animals per genotype). d) The graph shows the normalized average body area ratio of wild-type, *ire-1(ok799)*, *xbp-1(tm2482)*, *pek-1(ok275)*, or *atf-6(ok551)* adult worms, calculated as follows: average body area of worms grown on 15 mM HU (*n* = 3 experiments totaling >30 individual HU-treated animals per genotype)/average body area of worms of the same genotype on DMSO. e) The graph shows the fractions of wild-type, *ire-1(ok799)*, *xbp-1(tm2482)*, *pek-1(ok275)*, or *atf-6(ok551)* worms grown past L4 stage on DMSO at 48 h posthatching (*n* = 3 experiments totaling >90 individual animals per genotype). f) The graph shows normalized fraction of wild-type, *ire-1(ok799)*, *xbp-1(tm2482)*, *pek-1(ok275)*, or *atf-6(ok551)* worms grown past L4 stage on 15 mM HU at 48 h posthatching, calculated as follows: fraction of worms past L4 on 15 mM HU/fraction of worms past L4 on DMSO (*n* = 3 experiments totaling >120 individual animals per genotype). In all graphs, the error bar represents standard deviation. Statistical analysis: ns *P* > 0.05, *P < 0.05, **P < 0.01, ****P < 0.0001 (ordinary 1-way ANOVA test corrected for multiple comparisons using Dunnett's method).

Because the UPR-ER is activated in somatic cells and embryos after *pri-1* or *pri-2* RNAi, we hypothesized that the UPR-ER protects worms from replication stress and the resulting DNA damage. To test this hypothesis, we used HU, a widely used chemical that inhibits ribonucleotide reductase, which reduces ribonucleosides into deoxyribonucleosides for DNA synthesis (Craig *et al*. 2012). In *C. elegans*, HU exposure leads to S-phase arrest, causing oversized nuclei in the mitotic germline, an extension of the duration of the first cell cycle in early embryos, and germline apoptosis (MacQueen and Villeneuve 2001; Garcia-Muse and Boulton 2005; Stevens *et al*. 2016). To quantify functional requirements of UPR-ER genes in response to replication inhibition, we compared the number of eggs laid per HU-exposed worm during a 4-h recovery period to the number of eggs laid by an unstressed worm of the same genotype, and also after prolonged replication stress by chronic HU exposure from the L1 stage onward. Acute and prolonged HU exposure both caused fecundity defects, but neither was exacerbated in the tested UPR-ER gene mutants (Fig. 6, a and b). Therefore, the UPR-ER is apparently dispensable to protect the *C. elegans* germline from replication stress.

### *ire-1* and *pek-1* are required to protect the soma from HU-induced replication stress

To test whether the UPR-ER protects somatic growth and development from damage caused by prolonged HU exposure, we measured the body area of worms grown on 15 mM HU from the L1 stage for 72 h. Because *ire-1* and *xbp-1* mutant worms have a smaller body size than wild-type worms (Fig. 6c), we normalized body size within each genotype (stressed/unstressed condition). We found that *ire-1* and *pek-1* mutant worms showed a reduced average body size ratio when exposed to HU (Fig. 6d). This suggests that the *ire-1* and *pek-1* branches of the UPR-ER are required for worms to tolerate or resolve prolonged replication stress to achieve normal somatic growth, while the *atf-6* branch is dispensable.

In addition to body size, we also quantified developmental success of worm mutants in the absence of stress and following HU exposure. Loss of *ire-1* caused developmental delay in unstressed conditions, whereas loss of other UPR-ER components did not (Fig. 6e). When exposed to 15 mM HU from the L1 stage on, *ire-1* or *pek-1* mutant worms showed a reduced ability to progress past the L4 stage within 48 h, whereas *atf-6* or *xbp-1* mutation had no effect (Fig. 6f). Collectively, these data show that the *ire-1* and *pek-1* branches, but not the *atf-6* branch, are required to maintain somatic resistance to replication stress.

### Transcriptome analysis of *pri-1* RNAi-treated embryos suggests deregulated glycosylation, calcium signaling, and fatty acid desaturation as potential sources of ER stress

**Fig. 7.**
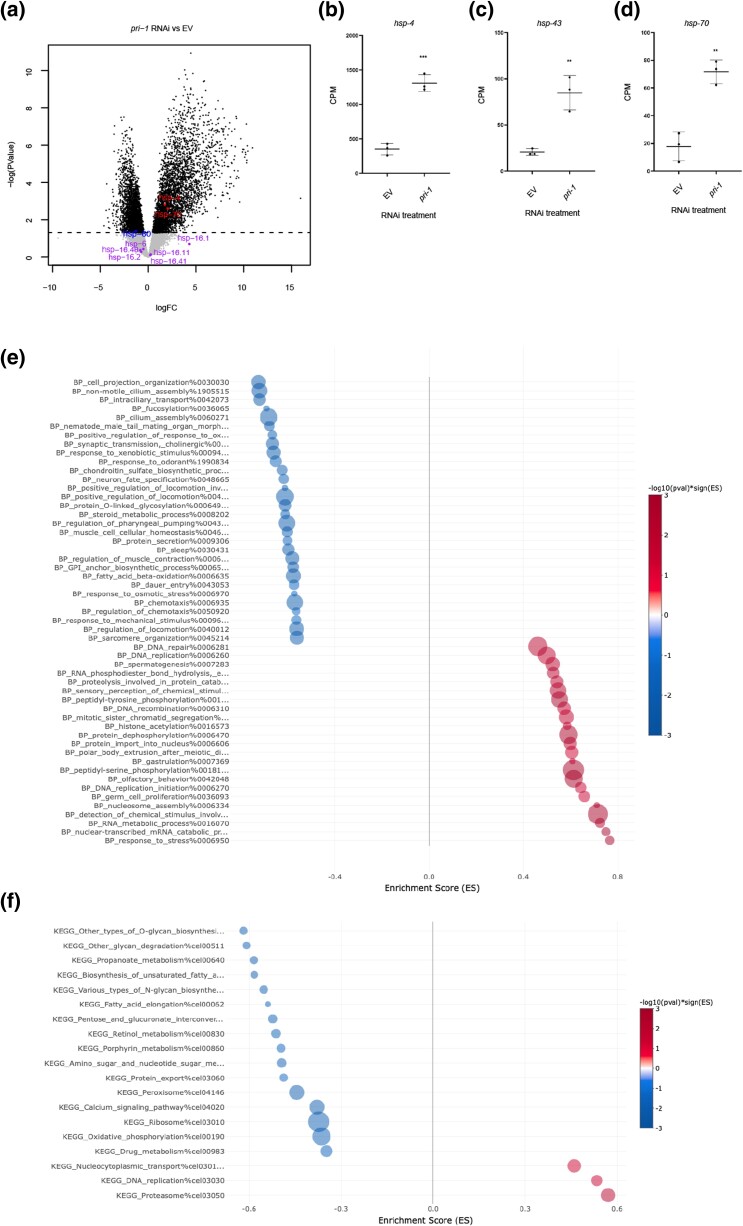
Replication stress in embryos alters protein glycosylation, calcium signaling, and fatty acid desaturation. a) The volcano plot shows the expression of all detected genes in EV and *pri-1* RNAi-treated embryos. X-axis, logFC; Y-axis, -log_10_(P-value). Black, *P-*value <0.05; gray, *P*-value ≥0.05; blue, highlighted and significantly downregulated; red, highlighted and significantly downregulated; purple, highlighted but not significant. b–d) The graph shows average transcript levels in counts per million (CPM) of *hsp-4*, *hsp-43*, and *hsp-70* mRNA in EV or *pri-1* RNAi-treated embryos; the error bars represent standard deviation (*n* = 3 experiments). Statistics: **P < 0.01, ***P < 0.001 (unpaired Student's t-test). e and f) The bubble plots show processes enriched negatively (blue, enrichment score <0) and positively (red, enrichment score >0) in *pri-1* RNAi-treated worms, based on the BP (e) and KEGG (f) databases. Bubbles represent the top 30 or fewer gene sets determined to be statistically significant (cutoffs *P* < 0.05, *P*adj < 0.25), as determined by analysis with the easyGSEA function of the eVITTA webserver (Cheng *et al*. 2021). The size of the bubble corresponds to the number of genes represented in each gene set. X-axis: enrichment score (ES).

To identify genes and processes altered by replication fork stalling, we studied the transcriptomes of wild-type embryos treated with EV or *pri-1* RNAi using RNA-seq. We identified 2785 genes that were up- and 1738 genes that were downregulated following *pri-1* depletion (Fig. 7a; Supplementary Tables 1–3 and Fig. 7). In line with the abovementioned data, *hsp-4* was significantly induced following *pri-1* depletion, as were two other chaperones, *hsp-43* and *hsp-70* (Fig. 7, b–d; Supplementary Tables 1 and 2); others have reported that *hsp-70* is induced by tunicamycin in an *xbp-1*-dependent fashion (Urano *et al*. 2002; Lim *et al*. 2014), suggesting that it is an effector chaperone of the UPR-ER. By contrast, neither the mitochondrial UPR chaperones *hsp-6* and *hsp-60*, nor any of the cytoplasmic UPR chaperones of the *hsp-16* family (*hsp-16.1*, *hsp-16.11*, *hsp-16.2*, *hsp-16.41*, and *hsp-16.48*) were induced (Supplementary Tables 1–3 and Fig. 8; Fig. 7a). Hence, unbiased transcriptome profiling confirms that the UPR-ER is specifically activated in embryos experiencing replication fork stress, whereas other UPRs are not.

To delineate how replication fork stress could induce the UPR-ER, we performed GSEA using the BP and KEGG databases. As expected, terms relating to the UPR-ER stress response were enriched, e.g. the terms “BP_response_to_stress%0006950” (which includes *hsp-4* and *hsp-70*) and “BP_PERK-mediated_unfolded_protein_response%0036499” (Fig. 7, e–f, Supplementary Tables 4 and 5). Furthermore, we observed an enrichment of terms related to DNA replication and DNA repair (Fig. 7, e and f, Supplementary Tables 4 and 5), as expected in worms experiencing replication stress. For example, “KEGG_DNA_replication%cel03030” was one of only three upregulated terms when using the KEGG database for analysis, while upregulated terms identified with the BP database included terms such as “BP_DNA_replication_initiation%000627”, “BP_DNA_repair%0006281”, “BP_DNA_recombination%0006310”, and “BP_double-strand_break_repair_via_homologous_recombination%0000724”. Finally, our analysis identified several processes whose downregulation could indicate the source of UPR-ER activation in *pri-1* RNAi-treated worms. Specifically, when using analysis with the KEGG database, of the only 13 downregulated terms, three relate to protein N- and O-glycosylation (“KEGG_Various_types_of_N-glycan_biosynthesis%cel00513”, “KEGG_Other_glycan_degradation%cel00511”, “KEGG_Other_types_of_O-glycan_biosynthesis%cel00514”), 1 relates to calcium signaling (“KEGG_Calcium_signaling_pathway%cel04020”), and 2 relate to the biosynthesis of unsaturated fatty acids (“KEGG_Fatty_acid_elongation%cel00062”, “KEGG_Biosynthesis_of_unsaturated_fatty_acids%cel01040”). We conclude that the dysregulation of multiple cellular processes by replication fork stalling after *pri-1* RNAi likely activates the UPR-ER in the embryos.

## Discussion

Animals such as *C. elegans* consistently experience and must handle diverse stresses in their environment. Optimal adaptation to such insults requires the deployment of multiple response pathways, and recent studies have identified functional crosstalk between stress responses such as the UPR-ER and the DDR. Here, we show that replication fork stalling strongly induces two branches of the UPR-ER in *C. elegans* embryos. In turn, the UPR-ER is required to protect worms from the deleterious effects of stalled replication forks. Surprisingly, analysis of transcriptional reporters and transcriptome data suggest that it is primarily the UPR-ER that is induced by this stress, whereas other UPRs are only mildly activated. Our data suggest that replication fork stalling specifically causes ER dysfunction, possibly by disturbing cellular processes that are unique, or especially important, to ER function.

### Replication fork stalling selectively activates the UPR-ER

The UPR-ER, the cytosolic UPR, and the mitochondrial UPR are interconnected adaptive pathways that ensure homeostasis in the face of stress. Conditions that impair general protein folding such as oxidative stress and protein degradation defects induce all three UPRs in *C. elegans* (Rodrigues *et al*. 2011; Hou *et al*. 2014; Bartoszewska and Collawn 2020; Taylor *et al*. 2021). Here, we observed robust induction of 2 UPR-ER reporters by *pri-1* or *pri-2* RNAi or UV–C irradiation, but did not observe the induction of cytosolic or mitochondrial UPR reporters. This lack of induction was confirmed by our unbiased transcriptome profiling. Together, these data indicate that ER proteostasis or membrane lipid equilibrium, but not cytosolic or mitochondrial proteostasis, is disturbed by replication stress. This selectivity appears to rule out a mechanism whereby DNA replication fork stalling causes protein misfolding, aggregation, and proteotoxicity in all organelles. A clue regarding the specific mechanisms underlying UPR-ER activation was revealed by transcriptome profiling following *pri-1* depletion, which revealed dysregulated molecular processes and pathways with important links to the ER. These include protein glycosylation, which is important for the modification of secreted, ER-synthesized proteins; calcium metabolism, which is vital for protein folding in the ER and whose interference via thapsigargin is widely used to study ER protein processing; and fatty acid desaturation, which is essential for maintaining normal ER membrane lipid composition and is monitored by the UPR-ER (Hou *et al*. 2014; Denzel and Antebi 2015; Burkewitz *et al*. 2020; Ho *et al*. 2020; Deng *et al*. 2021; Xu and Taubert 2021). None of these processes are known to impact the activity of the cytoplasmic or mitochondrial UPRs, which may explain why replication fork stalling selectively activates the UPR-ER. However, it remains unclear why genes in these pathways are dysregulated by replication fork stalling.

The outer nuclear membrane is continuous with the ER and linked to the lumen of the ER, suggesting that ER stress responses may be directly linked to disturbances in nuclear processes such as DNA replication and transcription. For example, UPR-ER activation indirectly helps removing stalled replication protein complexes, and thus restarts replication by helping replication fork turnover; although such a mechanism may be less relevant in UV–C-treated animals with DNA damage, it could be especially relevant in animals with reduced primase complex activity.

### Replication stress induces noncanonical, *atf-6*-dependent *hsp-4* expression in embryos

The *hsp-4p::gfp* reporter is widely used in *C. elegans* to monitor the activity of the UPR-ER and to infer the presence of ER stress (Calfon *et al*. 2002; Urano *et al*. 2002; Ho *et al*. 2020). Canonical activation of this reporter depends strictly on the IRE-1-XBP-1 pathway. Here, we observed only partially *ire-1*- and *xbp-1*-dependent activation of *hsp-4* after *pri-1* or *pri-2* depletion in embryos. This is surprising because IRE-1 is the only known UPR-ER sensor that processes the unspliced *xbp-1u* mRNA into the mature *xbp-1s* product, which is then translated into XBP-1, the transcription factor that upregulates *hsp-4* expression (Walter and Ron 2011; Gardner *et al*. 2013; Senft and Ronai 2015; Kopp *et al*. 2018; Hetz *et al*. 2020; Xu and Taubert 2021). Because ATF6 is required to express XBP1 mRNA in humans, we studied *atf-6*, which represents the third branch the UPR-ER in *C. elegans* but is thought to be largely dispensable in this organism for stress-induced UPR-ER activity, as many ER stress-activated genes do not require *atf-6* for induction (Shen *et al*. 2005; Lee *et al*. 2007). Interestingly, *atf-6* loss of function significantly diminished *pri-1* or *pri-2* RNAi-induced *hsp-4p::gfp* activation in embryos. In somatic cells, transient *hsp-4* induction that is independent of *ire-1* and *xbp-1* occurs during the differentiation of stem-like seam cells into alae-secreting cells (Zha *et al*. 2019). Although the transcriptional factor B-lymphocyte–induced maturation protein 1 (*blmp-1*) is required to suppress *hsp-4* in this context (Zha *et al*. 2019), how it is activated is unknown. Our data suggest that *atf-6* may be involved in this process, in line with the view that *atf-6* plays important roles in *C. elegans* development. In sum, our data identify an important new nuance of the mechanisms that fine-tune UPR-ER activation in *C. elegans*.

### *ire-1* and *pek-1* are required for resistance to replication fork stress

A bidirectional crosstalk between the DDR and the UPR-ER has begun to emerge (González-Quiroz *et al*. 2020; Bolland *et al*. 2021). Yeast IRE1 is required for survival on HU (Zha *et al*. 2019). We found that the IRE-1 branch is required to protect *C. elegans* during prolonged HU exposure initiated at an early developmental stage. By contrast, short-term acute HU exposure at a later developmental stage was tolerated, similarly to what has been reported about treating *ire-1* worms at L4 stage with *rad-51* RNAi to induce DNA damage (Levi-Ferber *et al*. 2014).

Little evidence exists for the roles of the other two branches in response to genotoxicity. We report here that the PEK-1 branch is activated by replication fork stalling and that *pek-1* is required for somatic resistance to HU. By contrast, the *atf-6* branch was not required to protect the soma or germline from HU. This was surprising because, as noted above, *atf-6* is required to induce *hsp-4* in embryos. Nevertheless, our data implicate the UPR-ER as a whole in response to replication fork stalling. Future work will be required to define *ire-1*- and *pek-1*-dependent processes that promote survival and growth in genotoxic conditions.

## Data Availability

The data described in this study are available in the main manuscript, the Supplementary material, or in a public repository. Supplementary Figs. 1, 3, 4, 5, and 6 describe additional experiments using GFP reporters, Supplementary Fig. 2 contains additional repeats of immunoblots, and Supplementary Figs. 7 and 8 provide additional description of the RNA-seq analysis. Supplementary Tables 1–3 contain lists describing gene expression data identified by RNA-seq; Supplementary Tables 4–5 contain lists describing processes identified by RNA-seq analysis. Supplementary material is available Xu J, Sabatino B, Yan J, Ermakova G, Doering KRS, Taubert S; 2023; Supplemental Material for Xu et al., 2023; Figshare: https://doi.org/10.25387/g3.24941133. Raw and processed RNA-seq files have been deposited: Sabatino B, Xu J, Taubert S; 2023; Effect of pri-1 (DNA primase) RNAi on gene expression in embryos of Caenorhaditis elegans; Gene Expression Omnibus (https://www.ncbi.nlm.nih.gov/geo/); GSE225569. See the methods for information on reagents and strains. *C. elegans* strains described for the first time in this study can be requested from the authors.
